# The Representation of Objects in Apraxia: From Action Execution to Error Awareness

**DOI:** 10.3389/fnhum.2016.00039

**Published:** 2016-02-10

**Authors:** Loredana Canzano, Michele Scandola, Valeria Gobbetto, Giuseppe Moretto, Daniela D’Imperio, Valentina Moro

**Affiliations:** ^1^IRCCS Santa Lucia FoundationRome, Italy; ^2^Department of Psychology, La Sapienza UniversityRome, Italy; ^3^NPSY-Lab.Vr, Department of Philosophy, Education and Psychology, University of VeronaVerona, Italy; ^4^UOC Neurology A, Azienda Ospedaliera Universitaria IntegrataVerona, Italy

**Keywords:** objects in apraxia, action recognition, imitation and pantomime, error awareness

## Abstract

Apraxia is a well-known syndrome characterized by the sufferer’s inability to perform routine gestures. In an attempt to understand the syndrome better, various different theories have been developed and a number of classifications of different subtypes have been proposed. In this article review, we will address these theories with a specific focus on how the use of objects helps us to better understand upper limb apraxia. With this aim, we will consider transitive vs. intransitive action dissociation as well as less frequent types of apraxia involving objects, i.e., constructive apraxia and magnetic apraxia. Pantomime and the imitation of objects in use are also considered with a view to dissociating the various different components involved in upper limb apraxia. Finally, we discuss the evidence relating to action recognition and awareness of errors in the execution of actions. Various different components concerning the use of objects emerge from our analysis and the results show that knowledge of an object and sensory-motor representations are supported by other functions such as spatial and body representations, executive functions and monitoring systems.

## Apraxia: A Multifaceted and Complex Syndrome

The term *Apraxia* covers a wide spectrum of disorders, all referring to motor cognition and the inability to perform actions that have been previously learned and/or were possible before the onset of the syndrome. These deficits cannot be explained by elementary motor or sensory deficits and are not due to language comprehension disorders (Zadikoff and Lang, 2005). Apraxia is usually the result of left frontal and parietal lesions (prevalence ranging from 28 to 57%, Donkervoort et al., 2000), although in some cases apraxia following right brain damage has been reported (prevalence ranging from 0 to 34%, Donkervoort et al., 2000). In addition, lesions involving the corpus callosum cause unilateral left apraxia. Thus, the left hemisphere appears to be dominant in processing actions (Petreska et al., 2007).

Apraxia is characterized by an automatic—voluntary dissociation (De Renzi et al., 1982). In other words, patients can execute spontaneous gestures when the environmental context induces their involuntary/automatic response (e.g., waving their hand to say goodbye when they are going away) but they are not able to intentionally execute the same action out-of-context or when asked to do so by an experimenter. For this reason apraxia is considered as a disorder of the voluntary and aware ability to perform gestures (Wolpe et al., 2014).

Steinthal first introduced the term *Apraxia* (literally = *without action*) in 1871 to describe the difficulty that certain patients had when they tried to execute an action which involved an object or a tool. He suggested that the deficit depends on disorders in the relationship between the patients’ movements and their abilities to manipulate objects (Steinthal, 1871, 1881). Since then, various different forms of apraxia have been described, some which involve objects, others which do not.

Liepmann (1920) proposed a classification of the different subtypes of apraxia with the aim of identifying the various motor and cognitive aspects. He identified three different subtypes. A person who is able to name familiar tools and objects but is almost totally unable to use them correctly suffers from *Ideational* apraxia. In this case, the person has lost the ability to conceptually organize intended actions. *Ideo-kinetic* (or ideo-motor) apraxia is a disorder affecting the production component of the praxis system, resulting from an apparent dissociation between the idea of an action and its execution. This also involves an inability to pantomime actions or mimic an action with an object or tool (without actually holding the object in question). Finally, *limb-kinetic* apraxia refers to a loss of dexterity or deftness, characterized by hesitations and a disrupted smoothness in movements (Liepmann, 1920; cited in Goldenberg, 2013).

**Figure 1:**
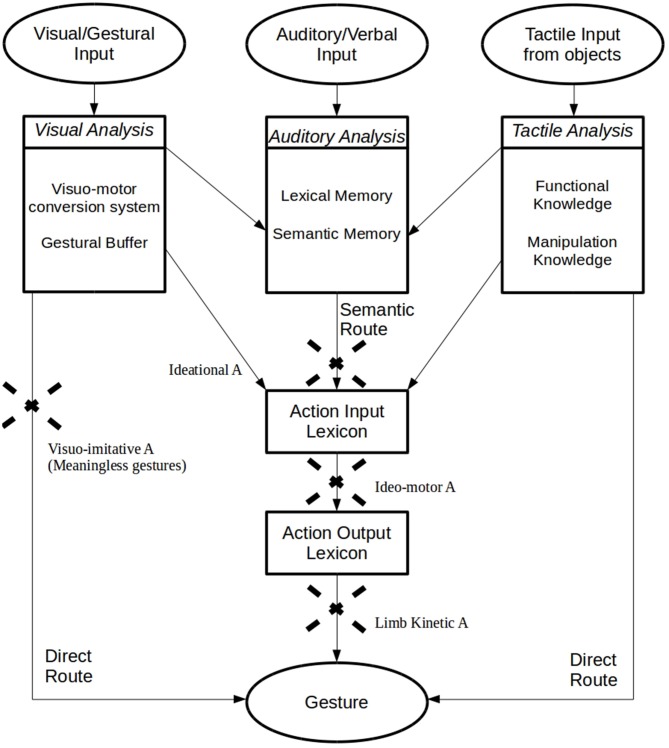
**Graphical representation of the “Dual-Route Model” (Gonzalez Rothi et al., 1991), with the additional proposal for the Visuomotor Conversion System, Gestural Buffer (Cubelli et al., 2000), Functional and Manipulation Knowledge (Roy and Square, 1995) and the Selective Tactile Route (Graham et al., 1999)**.

The cognitive nature of apraxic deficits was also discussed by Geschwind who suggested that apraxia does not extend to novel or meaningless movements, but exclusively concerns learned motor skills: “*the hemisphere dominant for handedness is a storehouse of the learning involved in the acquisition of motor skills*” (Geschwind and Damasio, 1985, p. 191). When this storehouse, localized in the lower left parietal area, is damaged or disconnected from verbal and visual commands or from the premotor cortex (Heilman et al., 1982; Petreska et al., 2007), patients are apraxic. However, this hypothesis was not exhaustive. Indeed, evidence of deficits in the imitation of novel, meaningless gestures (with meaningful actions spared) has led to the identification of a new subtype of apraxia, the *visuo-imitative apraxia* (Goldenberg and Hagmann, 1997). In this case, patients do not present with a general defect affecting imitation but suffer from a specific deficit in the imitation of meaningless gestures. This dissociation has been explained by the Dual-Route Model (Gonzalez Rothi et al., 1991) that suggests the existence of two streams involved in the production and imitation of actions. With the *direct route (*or *non-lexical route)*, the gesture is produced by means of a direct translation of visual input into motor outputs. This permits the imitation of both novel, meaningless gestures and significant and familiar actions. The alternative, *semantic route* (or *lexical route)* needs lexical, semantic memory and is exclusively useful for familiar and meaningful gestures (Gonzalez Rothi et al., 1991). Thus, an interruption in the direct route does not affect meaningful actions, but it does cause a specific disorder affecting the selective imitation of new and meaningless gestures (visuo-imitative apraxia). The Dual-Route Model was revised by Cubelli et al. (2000) who added a system specifically devoted to the direct transcoding of visual input into motor programs (the “visuo-motor conversion mechanism”) and a system for short-term representation of the whole action (the “gestural buffer”; Figure 1).

Taking all these approaches to apraxia into account, we may consider that both sensory–motor and cognitive components play a role in the execution of gestures (Buxbaum et al., 2014; Goldenberg, 2014; Osiurak and Le Gall, 2015). Using an object or pantomiming the use of an object without a model certainly requires the recruitment of cognitive functions such as knowledge of the object and its function and/or the context in which it is usually employed. Nevertheless, these need to be integrated with motor and sensory functions (Goldenberg, 2013, see below). Body representations may also play a crucial role. A strong connection between gestures and the body has been demonstrated in apraxia. Goldenberg (1995) have shown that knowledge of one’s own body is necessary in gesture imitation. Indeed, patients who are impaired when executing gestures involving their own body are also impaired when reproducing the same gestures using a manikin. This indicates a close link between body representations and action planning. Disorders in imaging and planning the functional relationship between body parts and objects are also suggested by the typology of the errors which apraxic patients frequently commit when they pantomime transitive actions: they often use their hand as if this was the object or a part of the object (Body part as object). Finally, the existence of effector-specific forms of apraxia suggests a relationship between body representations and gesture making disorders. In fact, various different types of apraxia have been described involving the face (upper/lower face apraxia, oral apraxia, orofacial apraxia, apraxia of speech), the eyes (eyelid apraxia, ocular apraxia, gaze apraxia), the limbs (hand apraxia, finger apraxia, apraxic agraphia, dressing apraxia, magnetic apraxia), the legs (leg apraxia, gait apraxia) and the trunk (axial apraxia; Petreska et al., 2007). These subtypes correspond to at least partially different lesion sites. For example, while an impairment in the imitation of hand gestures is associated with left inferior parietal lesions, impairments in the imitation of finger gestures may follow both right and left pre-central and inferior frontal lesions. Disorders in lower face movements are a consequence of damage to the left ventral precentral frontal gyrus, while deficits in upper face movements may follow both left and right sided lesions (Goldenberg, 2013). In this article, the term apraxia will refer to limb apraxia unless otherwise specified. In general, limb apraxia is more frequent after left as compared to right hemisphere brain damage. In addition, left hemisphere lesions usually cause bilateral signs of apraxia, while damage to the right hemisphere only affects the left hand (Petreska et al., 2007).

The role of body representations in the execution of gestures was also suggested by Buxbaum et al. (2000) in their revision of Rothi’s model. The authors emphasized the importance of spatial components, in particular the need for updates regarding the reciprocal spatial dynamic positions of the body parts in relation to an object while an action is being executed. This stage of an action is between the lexical and non-lexical route and subserves both meaningful and meaningless actions.

In this context, apraxia appears to be a complex, multifaceted syndrome. In addition to specific knowledge (of an object, its function and its relative context) and sensory-motor abilities (the planning and execution of actions), other elements such as body and space representations may affect the execution of a gesture. As such, understanding the role of objects in apraxia may help us to achieve a better understanding of the nature of apraxia. Indeed, deficits related to the use of objects have been recognized as the main symptom of the syndrome since it was first identified (Steinthal, 1871) and it is this disorder that patients complain about most. Moreover, research has recently shown that the shape and position of objects can activate motor responses in healthy people (*affordance*, Gibson, 1979; Ellis and Tucker, 2000) and that body-object interaction may involve specific non-semantic types of knowledge (e.g., memory of movements, knowledge regarding manipulation, mechanical problem solving, action monitoring and error awareness).

The role of objects in action execution and error recognition is the topic of this article. We will start by describing some types of apraxia and the main dissociation between deficits involving the use of objects (i.e., transitive actions) and those which do not involve objects (i.e., intransitive actions). We will also briefly introduce two specific subtypes of apraxia that in some way involve the incorrect use of objects: constructional apraxia (Critchley, 1953) and magnetic apraxia (Denny-Brown, 1958). Deficits in action execution will then be analyzed with reference to the three tasks usually administered in the assessment of apraxia: the use of objects and the imitation and pantomiming of actions. Finally, the potential effects of apraxia on the recognition of actions and the role of objects in the detection of errors in the execution of actions will be discussed.

## Objects in Apraxia

The distinction between transitive and intransitive gestures is based on whether or not an action involves the use of an object. The transitive/intransitive dissociation has been documented in several case studies reporting gesture-specific forms of apraxia (Rapcsak et al., 1993; Dumont et al., 1999).

A transitive gesture is tool-based (e.g., hammering in a nail) and it is in some way shaped by the nature of the object and by any knowledge possessed regarding its functions or potential uses. Indeed, if an object is actually present, the action may take a third route, in addition to the two previously mentioned routes in Rothi et al.’s (1985) model, the “selective tactile route”. There is evidence that this third route may be specific and crucial for actions involving objects, which may potentially be driven by tactile information inherent to objects (Graham et al., 1999). The existence of this additional route seems to be confirmed by evidence collected from a patient who was impaired when responding to verbal or visual commands which requested him to perform certain gestures, but who was able to execute an action when he took hold of a tool (Buxbaum et al., 2000). In spite of this “third route”, accuracy in transitive gestures is usually reported to be lower than in intransitive gestures (Haaland and Flaherty, 1984; Gonzalez Rothi et al., 1988; Schnider et al., 1997; Haaland et al., 2000), although apraxic patients are often impaired in both transitive and intransitive actions. Nevertheless, it has been suggested that symptoms affecting both hands only affect transitive gestures, while disorders in intransitive actions usually only involve the contralesional hand (Watson et al., 1986; Binkofski et al., 2001).

A problematic aspect concerning the classification of transitive actions concerns the distinction between single step and multiple step gestures. Some authors (Heilman and Rothi, 1993; Raymer and Ochipa, 1997) have suggested a distinction between the difficulty experienced when using a tool or object (conceptual apraxia) and the inability to execute multistep actions (ideational apraxia). Of course, both of these involve the idea of an action, but in the case of ideational apraxia, errors might be due to other than sensory-motor errors (e.g., step omissions or perseverance). For this reason, the definition relating to Action Disorganization would seem to be more appropriate (Schwartz et al., 1995; Humphreys and Forde, 1998). Action Disorganization refers to cases where habitual actions are performed perfectly but disturbances arise when an action requires a preformed plan in accordance with a specific goal (Poeck, 1983; Schwartz et al., 1993, 1995; Goldenberg et al., 2007). In this way, we can see it is possible to distinguish between ideational components and more executive aspects.

An important issue in the debate on transitive actions concerns the source of the knowledge which is necessary for the appropriate use of an object. Two types of knowledge are considered to be necessary: knowledge regarding the features relating to a particular tool or object (Functional Knowledge) and knowledge of the action required for that object and of how to organize the individual motor sequences involved in that action (Manipulation Knowledge; Roy and Square, 1995).

Functional knowledge of tools lies in the semantic memory (Goldenberg and Randerath, 2015) and associates various types of tools with their purpose and the actions they can be used for. When a tool has several possible uses, functional knowledge is used to weigh these up based on their relative frequency and familiarity. The prototypical use invariably predominates (Goldenberg, 2013).

Manipulation knowledge refers to the (modality specific) motor representations that underlie the use of familiar tools and objects. This corresponds to the “engrams” or “movement memory” (Heilman and Rothi, 1993) that are thought to contain the features of gestures (i.e., muscular and joint actions, hand postures) which are invariant and critical when one needs to distinguish between one gesture and another (Buxbaum, 2001). However, each action requires adaptations of its invariant features in order to deal with changes in environmental conditions (e.g., the position, shape or size of an object). These engrams cannot therefore be rigid and stable. Goldenberg (2013) suggests that this specific manipulation knowledge is only necessary for the special, expert use of a tool (e.g., using a hammer for sculpting, playing a violin) but not for conventional tools. In everyday activities, manipulation knowledge would be replaced by the interaction between general functional knowledge and mechanical problem solving processes (Goldenberg, 2013, p. 125). Mechanical problem solving involves the ability to infer its function from the structure of an object (Goldenberg and Hagmann, 1998). It refers to general rules in the context of mechanical interactions with objects rather than to the functional properties of an individual object. These rules are based on the general principles of physics and mechanics that people acquire over the course of their lives (“folk physics”, Povinelli et al., 2000) and they apply to concrete constellations of tools and objects. As familiar and novel objects share a similar repertoire of functionally significant parts and properties (e.g., a handle, a blade) and since familiar applications of tools obey the same physical regularities, mechanical problem solving allows the accommodation of new objects and assists in the identification of alternative ways of using familiar objects (e.g., a coin used as a screwdriver). Deficits in functional knowledge lead to the defective use of common tools, while disorders in mechanical problem solving impact unusual, alternative uses of familiar objects and novel tools (Goldenberg, 2013). Of course, in both situations components relating to knowledge about an object, sensory-motor information and spatial and body representations are involved. However, while people exclusively rely on previously learned contents when using common tools, mechanical problem solving (which is necessary for novel actions) requires the integration of these components in a totally new way or in a way that is only partially similar to previously used methods. This may explain the fact that some patients can perform habitual actions but are totally unable to use unusual objects.

We can thus understand that when people perform new, unusual actions, the mechanical problem solving they resort to is based on information provided by the object they wish to use. When people identify an object, they activate exploratory movements to upload its tactile properties (Loeb and Fishel, 2014). “*Perception is not something that happens to us or in us: it is something we do*” (Noe, 2004, p. 1). An elegant exemplification of this affirmation was made by Gibson (1979) who coined the term “affordance”, that is, the implicit effect of the association of an object with the various actions and functions that it allows. Affordance depends on the setting between the physical properties of the body and the physical features of the environment (Warren, 1984; Adolph and Berger, 2006). Ellis and Tucker (2000), in fact, proposed the term “micro-affordances” to refer to the activation of action components appropriate for interacting with objects. The fact that body representations are necessary has been demonstrated in studies indicating that adults judge affordances with respect to intrinsic information about their bodies (Warren, 1984; Mark, 1987; Warren and Whang, 1987; Mark et al., 1990). However, it is not only the perception of affordance that guides an action: perception and actions are in a continuous feedback loop (Patla, 1998; Adolph and Berger, 2006; Franchak et al., 2010). In addition to the pragmatic process, which includes an analysis of the various different affordances and potential translations into action, higher order visual areas provide a perceptually based parallel semantic description of the object (Jeannerod et al., 1995; Ellis and Tucker, 2000; Maranesi et al., 2014).

Thus, using tools and grasping objects (with a configuration of the hand in accordance with the object) are highly specialized behaviors in primates (Jeannerod et al., 1995; Macfarlane and Graziano, 2009; Maranesi et al., 2014) indicating that they are able to reinterpret the physical world as a series of abstract features (Penn et al., 2008). An inability to use tools may thus reflect damage to the “stored representational system of gestures” (Buxbaum, 2001). This system supports representations regarding a tool (the Functional Knowledge), its associations and the purpose of any actions performed with it (the Manipulation Knowledge) or, as Luria (1978) suggested, it may be the result of deficits in executive planning (i.e., Dysexecutive syndrome). Finally, some authors attribute difficulty in using tools to a specific problem with technical reasoning (Gagnepain, 1990; Le Gall, 1998; Osiurak et al., 2009, 2010), including difficulties in identifying and unifying the technical means relevant for a given technical end (Jarry et al., 2013).

Taken as a whole, these complementary analyses of the various processes involved in transitive actions make it possible to identify a further component, a sort of implicit, non-verbal, practical/technical reasoning which may or may not be dissociable from executive functions. Although Mechanical Problem Solving is based on all the other components (the visual and tactile perception of objects and environments, motricity, spatial and body representations), it is probably crucial to understanding apraxia (Goldenberg, 2013). For example, it may explain two well-known dissociations: the automatic/voluntary association and the know/unknown action dissociation. Ignoring these aspects often leads to an underestimation of any diagnosis of apraxia in patient reports and the onset of symptoms is only reported after the patient has been discharged from hospital.

We also wish to put forward a hypothesis suggesting errors linked to the various components of the execution of an action may be differently associated with Functional Knowledge or Mechanical Problem Solving.

Several classifications of apraxic errors in the use of objects have been suggested (De Renzi and Lucchelli, 1988; Humphreys and Forde, 1998; Schwartz et al., 1998; Goldenberg et al., 2001; Rumiati et al., 2001; Petreska et al., 2007). Among these, errors due to disorders in Functional Knowledge may be Perplexity, Conduits d’approche, Omission and Misuse (involving content, substitutive, augmentative, fragmentary and associative errors, Petreska et al., 2007).

Patients who show Perplexity seem to have no idea what they can do with an object: “*The patient looked hesitatingly at the objects, picked up one of them, turned it over, put it down, then tried with another object, giving unmistakable signs of not knowing what to do*” (De Renzi and Lucchelli, 1988, p. 1177). Sometimes patients seem to try various different actions in order to progressively reach the right one (e.g., when trying to use a toothbrush, the patient starts hitting his/her cheek, reaches his/her mouth and is finally able to brush his/her teeth). These Conduits d’approches are very similar to those of aphasic patients when speaking. Omissions may be present in multistep actions such as when patients forget “*to carry out an action necessary for completing the sequence, for example, the stamp was not moistened*” (De Renzi and Lucchelli, 1988, p. 1177) and this leads to incomplete executions. In the case of Misuse, the object is used in a conceptually inappropriate way or is used as if it was another object (Parapraxic errors). Here the patient not only does not have any idea of what to do, but seems not to realize his/her difficulty when using the object incorrectly. Other errors indicating object misuse are the replacement of one movement with another that shares one or more similar features (Associative errors), the fragmentation of gestures or the production of inappropriate steps (Augmentative errors).

The disorders which are linked to Mechanical Problem Solving seem to be Clumsiness, Mislocation, Sequence errors and Perseveration. Clumsiness refers to when an action appears to be conceptually correct for the tool but is “*carried out in an awkward and ineffectual way, because of poor control of skilled hand movements*” (De Renzi and Lucchelli, 1988, p. 1177). Mislocation is when an object is used in an appropriate way but in a non-appropriate place. Spatial misorientation of an object or of an object with respect to the body is also sometimes considered to be the same type of error. When the Sequence is incorrect, part of an action is executed without the previous step having been completed (e.g., the envelope is sealed before the letter is placed inside it). Finally, Perseveration refers to a situation where a patient continues to repeat part of an action without any apparent aim or he/she is unable to stop executing one step in order to execute the next.

Although these errors are much more frequent in left damaged apraxic patients, it is worth noting that very similar errors may be also present in non apraxic patients. For example, Mislocation and errors in Trajectory are frequent in right hemisphere damaged people (in particular in the presence of spatial neglect) and Perseveration and Frequency errors are a typical index of frontal damage. Although the most part of right hemisphere damaged patients’ errors are usually considered due to spatial and more general attentive disorders (Goldenberg, 2013), only in depth qualitative investigation will enable a better understanding of the various different expressions of action errors.

## Constructional Apraxia

Constructional apraxia was defined by Benton as “*the impairment in combinatory or organizing activity in which details must be clearly perceived and in which the relationship among the component parts of the entity must be apprehended*” (Benton, 1967). Although constructional apraxia is usually assessed by means of drawing or copying tasks, this also impacts the patient’s ability to put together the components of an object (e.g., a coffee machine or a food mixer) with consequences affecting everyday activities. The main cause of this syndrome seems to involve a disorder in Mechanical Problem Solving. Nevertheless, other action components may impact on constructional abilities. Critchley (1953, p. 191) described this form of apraxia in these terms: “*The defects which characterize constructional apraxia essentially involve those movements which are directly concerned with space *per se*, i.e., manipulation of the three dimensions of space, and particularly the translation of an object from one spatial dimension into another*”. In fact, lesions in both the right and left hemisphere may produce constructional apraxia, although the symptoms are qualitatively different. After left hemisphere lesions, errors regard the comprehension of the function of an object or its parts, the sequence required to put together the various parts and the organization of that sequence. Copies of drawings respects the appropriate distance to the model and the global orientation and outlines, although the drawing appears impoverished by lack of or simplification of details. In contrast, in the case of right hemisphere lesion, patients mainly commit spatial errors regarding the positioning of the individual parts of an object and their reciprocal relations. Copies of drawings are badly placed and sometimes too close to the model or overlapping (“closing in”) with a distortion of the horizontal and vertical axes (Goldenberg, 2013). When spatial neglect is present, the parts of the picture in the contra-lesional space are totally omitted and the global structure is broken.

## Magnetic Apraxia

The compulsive tactile exploration and object grasping which often occurs in the contra-lesional hand after left or right frontal lobe damage is called Magnetic Apraxia (Denny-Brown, 1958; Moro et al., 2015). In this condition, the mere visual presence of an object near the hand (or touching the hand) triggers groping movements as well as grasping. In spite of the fact that these movements appear to be goal directed, they are totally involuntary and the patient is not able to inhibit the behavior of the hand. Magnetic Apraxia is often associated with grasping, an inability to release the grip (Forced grasping response) and groping (i.e., movements toward a stimulus based on the mere proximity of the stimulus and not triggered by tactile stimulation). In addition, utilization behavior (i.e., involuntary and inappropriate use of objects) and the compulsive involuntary manipulation of tools may be present. Finally, when magnetic apraxia is a symptom of the Anarchic Hand syndrome, it may be associated with Intermanual conflict (i.e., the hand movements interfere with non-anarchic actions) and Diagonistic dyspraxia (i.e., uncontrolled cross-purpose actions of the Anarchic Hand are triggered by voluntary activities of the non-Anarchic Hand; Moro et al., 2015).

These involuntary movements may lead one to object that Magnetic Apraxia is not strictly a form of apraxia. In fact this is not a disorder affecting the voluntary and aware ability to make gestures (Wolpe et al., 2014). Nevertheless, alterations in object-body (i.e., hand) interactions are the main symptom of Magnetic Apraxia associated with an inability to inhibit involuntary actions and the exacerbation of automatic responses. The result is a dysfunctional use of objects.

## Imitation and Pantomime

Despite the fact that early descriptions of limb apraxia mainly concerned the difficulty that patients experienced in the use of objects, assessments of apraxic symptoms are usually carried out by means of imitation and pantomime tasks (for the object use task, see De Renzi and Lucchelli, 1988). In imitation tasks, subjects are asked to reproduce the actions executed by the examiner, while in pantomime tasks they are requested to make specific gestures on verbal or kinaesthetic command or after an object is presented (but with the pantomime being performed without the object).

The differences between these two types of tasks are crucial if we wish to understand the nature of apraxia and the potential role of objects (Goldenberg, 2013). In fact, in a seminal model of apraxia, Roy and Hall (1992) proposed distinguishing between two sequentially arranged phases in gesture production. In the first phase, a mental image of the action is created using the long-term memory (with the involvement of the Semantic Route of Gonzalez Rothi et al., 1991). This is typical for pantomime tasks but not necessary for imitation tasks. In the second phase, the image is converted into motor response programs (in addition to all the components previously discussed). In imitation tasks, only this second phase is necessary since an image of the action is provided by the examiner who executes the gesture that patients have to imitate (the Direct Route, following Gonzalez Rothi et al., 1991). From this perspective, a deficit in imitation would always be associated with a disorder in pantomime due to the deficit affecting the second phase of the process which is common to both the tasks.

The fact that a process of action goal recall is possibly also involved in imitation tasks belies Roy and Hall’s theory. According to the Theory of Goal-Directed Imitation (GOADI; Wohlschläger and Bekkering, 2002), an imitator does not necessarily need to imitate the observed movement but can use the model as a cue to select pre-existing motor programs. In this case, when the gesture is executed, the motor program does or does not match the movement of the model, but the main goal of the action is achieved properly. The central principle of GOADI is that the selected goals elicit the motor program with which they are most strongly associated even though these motor programs may not necessarily lead to matching movements (Wohlschläger et al., 2003). In this way, the existence of two routes sustaining imitation is postulated, one of which relies on existing motor programs and the other that bypasses them. Only familiar actions can use pre-existing motor programs, while new unfamiliar gestures replicate the motor programs showed by the model. As a result, unfamiliar actions may be more similar to the model than familiar ones. Gravenhorst and Walter (2009) advance the idea that an interference effect of familiarity is modulated by perception, and that perception is in turn modulated by habitual style. Dissociations in the ability to imitate familiar and unfamiliar gestures are thus possible.

To sum up, both pantomime and imitation can be performed when action goal and motor memory recall come into play. Nevertheless, while imitation can be also performed without these elements, pantomime can not. Probably for this reason, it has been suggested that the most sensitive test in order to assess motor memory and action goal recall in apraxia is the pantomime on verbal command task since this provides the least cues and is almost entirely dependent on stored learned movement representations (visuo-kinesthetic movement engrams or *praxicons*, Mozaz et al., 2002). Seeing or holding a tool, as well as observing an examiner perform a pantomime, may provide a patient with cues, and if the movement representation is only partially degraded, these cues may obscure the diagnosis (Mozaz et al., 2002). In fact, as previously discussed, it has been suggested that not only visual, but also tactile feedback about the shape, weight and other properties of an object or tool may have a role in eliciting correct actions. In particular, De Renzi et al. (1982) showed that there are no differences in pantomime on verbal command with the tool in sight, while patients improve in the condition when, although blindfolded, they execute the action with the tool in their hand. Nevertheless, more recent evidence questions the possibility that tactile feedback *per se* is sufficient to evoke motor programs of correct tool use, and suggests that the facilitation is rather induced by the provision of additional information on the structural and functional features of the real use of the tool (Goldenberg, 2013) and all the possibilities that the environment offers (“affordance”, Gibson, 1979). The *“creation of pantomimes requires transformation of knowledge about the tool and its manipulation into empty-handed gestures that communicate the identity of the tool and the manner of its manipulation to other persons*” (Goldenberg, 2013, p. 155).

A qualitative analysis of the errors which occur in the pantomime of transitive gestures is particularly interesting as it provides evidence of a distortion in the body-object relationship. In fact, gestures may be correct in terms of the identification of the tool and the action, but patients fail because they use a body part as if it was the object (*Body part as object*, Goodglass and Kaplan, 1963) or demonstrate the shape of the object rather than pantomime its use (Goldenberg, 2013).

In their analysis of errors, Buxbaum et al. (2000) consider four components of gesture imitation and pantomime. These are: (i) Hand Posture/Grasp—i.e., when “*the hand posture/grasp is unrecognizable, flagrantly incorrect or transiently correct*; (ii) Arm Posture/Trajectory—i.e., when “*the arm posture and/or the trajectory/shape of the movement are flagrantly incorrect or only transiently correct*”; (iii) Amplitude of movement—i.e., when “*the size of the movement is clearly too large or too small, or the size is only transiently correct*”; and (iv) Timing/Frequency of movement—i.e., when “*the speed of the movement is flagrantly too fast or slow and/or the number of cycles is flagrantly too few or many*” (Buxbaum et al., 2007, p. 423; see also Buxbaum et al., 2000, 2005; Moro et al., 2008, 2015).

With the exception of one report (Belanger et al., 1996), many studies have reported that patients with apraxia are more impaired when performing transitive pantomimes (e.g., using a knife to cut bread) than intransitive gestures (e.g., waving goodbye; Haaland and Flaherty, 1984; Gonzalez Rothi et al., 1988; Roy et al., 1991; Schnider et al., 1997; Foundas et al., 1999; Haaland et al., 2000). Similar results have also been found in healthy people (Mozaz et al., 2002; Carmo and Rumiati, 2009). Although it is possible that the movements associated with transitive pantomimes are more complex than those involved in intransitive gestures, differences in the frequency with which these gestures are performed may also have a role. When people observe other people or when they want to communicate with a nonverbal message, they activate representations of intransitive gestures. In contrast, people primarily use transitive postures when they use tools or objects. A request to perform a transitive pantomime is thus less natural than a request to make an intransitive gesture (Mozaz et al., 2002).

At least partially different neural correlates have been reported between pantomime and imitation. The most common impairment after LBD involves both pantomime and imitation in both transitive and intransitive gestures, with more deficits for transitive than intransitive actions (Goodglass and Kaplan, 1983; Roy et al., 1993; Almeida et al., 2002; Stamenova et al., 2010). Conversely, selective deficits in imitation have been more frequently found after LBD for intransitive gestures. Disorders in imitation of of transitive gestures have been shown also after RBD, in both acute and chronic patients (Stamenova et al., 2010). Of course, given the role of the right hemisphere in spatial functions, these selective deficits may represent a secondary effect of deficits affecting the processing of visuo-spatial information or the translation of the spatial component of a movement into action (Roy, 1996).

Some authors have suggested that the left hemisphere may control transitive gestures while both hemispheres may be involved in the control of intransitive gestures (Haaland and Flaherty, 1984; Mozaz et al., 2002; Buxbaum et al., 2007). Nevertheless, more recent neuroimaging studies indicate that both transitive and intransitive gesture execution activates a common left hemisphere network involving frontal, parietal and temporal regions (Króliczak and Frey, 2009). This does not exclude a participation of the right hemisphere in the qualitative features of gestures. In fact, in spite of the dominance of the left hemisphere, the same studies have shown bilateral activation during preparation for pantomime performance (Króliczak and Frey, 2009) and during observation of actions (Grèzes and Decety, 2001). This involvement of both hemispheres in the control of movement may explain the rare cases of apraxia after right hemisphere damage.

**Figure 2:**
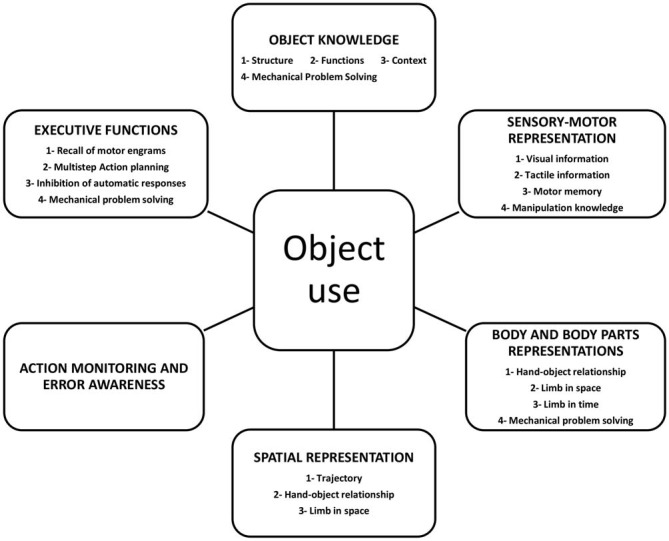
**Graphical representation of the components involved in object use**.

This analysis of studies which have specifically addressed components of object use, pantomime and imitation has provided evidence supporting the idea that using objects represents a complex function involving the integration of multiple components. A preliminary (but probably not exhaustive) representation of these components is shown in Figure 2.

## Action Recognition

The relationship between action execution and recognition has for some time been a matter of debate due to the inconsistent results from clinical and neuropsychological studies. The first clinical report indicating that patients with focal lesions could also have deficits in gesture recognition came at the same time as the first description of apraxia (Finkelnburg, 1870). A century later, in the 1980’s, a few pioneering studies on patients with limb apraxia reported an association between the inability to perform gestures and to understand their meaning and left parietal lesions (Heilman et al., 1982; Rothi et al., 1985; Watson et al., 1986). Since then, many studies have reported a co-occurrence of the two disorders (Duffy et al., 1975; Gainotti and Lemmo, 1976; Ferro et al., 1983; Duffy and Watkins, 1984; Rothi et al., 1985; Varney and Damasio, 1987; Wang and Goodglass, 1992; Bell, 1994; Buxbaum et al., 2005; Pazzaglia et al., 2008b) with the result that some authors describe ideomotor apraxia in these terms: *“These patients typically have no difficulty with object recognition, are deficient in performing skilled actions with objects, and even more tellingly, are impaired in recognizing object-related actions. The impact of ideomotor apraxia clearly extends beyond laboratory tasks. IMA patients make more errors with implements while eating than subjects without apraxia, and gesture recognition and tool manipulation knowledge are strongly significant, independent predictors of sequencing errors in multistep naturalistic action”* (Buxbaum and Kalénine, 2010, p. 203).

This very close link between the perceptual and the motor components of actions finds its neuronal correlates in the discovery of bimodal neurons and in particular of the mirror system (Gallese et al., 1996; Fogassi et al., 2005) where neurons are activated during both action execution and observation (Avenanti et al., 2007; Aglioti et al., 2008; Candidi et al., 2008; Sacheli et al., 2013; Tidoni et al., 2013; Urgesi et al., 2014). Results from neuropsychology, neuroimaging and electrophysiological studies based on the effects of temporary virtual lesions induced by repetitive transcranial magnetic stimulation demonstrated that this system and in particular the inferior frontal cortex is crucial for action understanding (Pobric and Hamilton, 2006), pure visual discrimination of actions (Urgesi et al., 2007; Moro et al., 2008) and imitation (Heiser et al., 2003).

Nevertheless, other studies involving a comparatively large sample of LBD and RBD patients, reported that those with left parietal and frontal lesions were impaired in gesture execution but failed to show any relationship between action execution and comprehension (Halsband et al., 2001; Negri et al., 2007). Some evidence was found regarding gesture recognition and the time course of a pathological process (acute vs. chronic; Ferro et al., 1983) and regarding the type and complexity of the gesture (Gainotti and Lemmo, 1976; Buxbaum et al., 2005). In addition, a number of neuropsychological single-case analyses report that the ability to imitate pantomimes is not necessary in order to be able to recognize object-associated pantomimes and the ability to use objects is not necessary in order to be able to recognize objects (for a review, see Negri et al., 2007). On the basis of these inconsistent results, it has been argued that motor production processes associated with object use are involved but not necessary for successful action or object recognition (Negri et al., 2007).

The idea of a complex multi-componential network involved in action recognition is also supported by some studies on lesions. Recognition deficits have been found to be correlated with both the left inferior parietal lobule (Buxbaum et al., 2005; Tessari et al., 2007) and the opercular and triangularis portions of the left inferior frontal gyrus (Pazzaglia et al., 2008b). Perception of different types of gesture may engage partially different networks. For example, Villarreal et al. (2008) pointed out that the right pre-supplementary motor area (pre-SMA), and bilaterally the posterior superior temporal cortex, the posterior parietal cortex, occipito-temporal regions and visual cortices are involved in the recognition of different types of gesture. This suggests that selective disruptions in different parts of the circuits may lead to distinct clinical deficits. Finally, Pazzaglia et al. (2008a) report neuropsychological evidence suggesting a close link between impairments in producing actions and impairments in recognizing the sounds of actions. The authors recruited two groups of patients (and a group of non-apraxic patients as the control), with bucco-facial and limb apraxia respectively. The first group was differentially impaired in imitating actions involving the mouth, while the other group (with limb apraxia) was differentially impaired in imitating actions performed with the hand or limb (e.g., using scissors). In a sound-picture matching task, the patients with (selective) bucco-facial apraxia failed to recognize mouth-related actions (e.g., slurping soup). In contrast, patients with (selective) limb apraxia were differentially impaired in sound-picture matching of limb-related actions. Both groups performed well in non-human related environmental sounds (e.g., an airplane flying).

Taken together, these results suggest that the perception, recognition, representation and execution of actions are heavily interactive processes in which various different features of the action (goal, meaning, kinematics, spatial organization, monitoring, etc.) co-operate. In this light, it might be simplistic to consider that a single lesional locus is responsible for all possible types of gesture recognition deficits and more in depth analyses are necessary.

Very recently a new aspect concerning the monitoring of action and the awareness of action-error has been investigated. Action and error monitoring are processes which have been well studied in the fields of psychology and neuroscience (for previous reviews, see e.g., Bush et al., 2000; Falkenstein et al., 2000; Taylor et al., 2007; Ullsperger et al., 2010). Specific but widespread brain areas are involved: the anterior insula, the anterior cingulate, the supplementary motor area, the thalamus, the brainstem, and the parietal lobe (Harsay et al., 2012). Electrophysiological and functional MRI studies have shown that our errors are processed as errors by the brain even if we are unaware of making them (Nieuwenhuis et al., 2001; Endrass et al., 2005, 2007; O’Connell et al., 2007, 2009; Pavone et al., 2009; Shalgi et al., 2009; Dhar et al., 2011; Hughes and Yeung, 2011). In particular, the anterior-cingulate region has been found to be associated with the generation of an electrophysiological pattern, Error-Related Negativity (ERN; Dehaene et al., 1994; Brázdil et al., 2002; Debener et al., 2005; Hester et al., 2005; Klein et al., 2007) that does not reveal any differences between aware and unaware errors (see also Stemmer et al., 2004). Nevertheless, awareness of errors is associated with larger bilateral activation of the prefrontal and parietal regions (Hester et al., 2005) or with left anterior insula activity (Klein et al., 2007).

When patients are able to identify and judge errors made by other people, but cannot recognize their own errors, they are considered to be affected by Anosognosia. Anosognosia can be defined as the impaired ability to recognize the presence of deficits in sensory, perceptual, motor, affective, or cognitive functioning or to appreciate their severity (Babinski, 1914; for a review, see Prigatano, 2010).

Among the various different types of anosognosia, the one which has been most investigated and is the most involved in action recognition is Anosognosia for Hemiplegia. In this condition, patients declare that they are able to execute actions with their paralyzed hand, to walk and to have an unrealistic degree of autonomy in daily life activities (Vocat et al., 2010; Moro et al., 2011). It has been suggested that this syndrome results from a combination of cognitive and sensorimotor dysfunctions, including impairments in the action monitoring system and in the detection of any mismatch between intention and outcome (Gandola et al., 2014; Preston and Newport, 2014). In fact, when forced to recognize their errors, at least some anosognosic patients improve their awareness (Fotopoulou et al., 2009; Besharati et al., 2015; Moro et al., 2015).

Although usually reported after right hemisphere damage, anosognosia for hemiplegia may also occur after left hemisphere lesion (Della Sala et al., 2009). So, the question is now whether a deficit in awareness may (or may not) exist in patients affected by apraxia and if so, how to distinguish it from a disorder in error monitoring.

In a recent study carried out by our group (Canzano et al., 2014), the first evidence for anosognosia in patients suffering from bucco-facial apraxia was found. Awareness deficits were considered to be present when patients showed that they were able to correctly evaluate the actions and errors made by other people but scored their own incorrect actions as being correct. This happened both in on-line judgement (i.e., at the moment of execution) and in off-line judgement, when patients watched themselves executing actions in a previously recorded video clip. In fact, in contrast with the ameliorative effects described in patients affected by Anosognosia for Hemiplegia (Fotopoulou et al., 2009; Besharati et al., 2015), self-observation by means of the video did not seem to impact the patient’s awareness of apraxic deficits. Previous studies have demonstrated deficits in action recognition in apraxic patients (Duffy et al., 1975; Gainotti and Lemmo, 1976; Ferro et al., 1983; Duffy and Watkins, 1984; Rothi et al., 1985; Varney and Damasio, 1987; Wang and Goodglass, 1992; Bell, 1994; Buxbaum et al., 2005; Pazzaglia et al., 2008a,b; Buxbaum and Kalénine, 2010). Nevertheless, this type of deficit was excluded in Canzano et al.’s (2014) study as a result of the patients’ spared ability to judge the actions of other people. The impact of these results is of course to date limited due to the low number of patients studied. Moreover, the results are currently limited to bucco-facial apraxia and would need to be verified for limb apraxia.

However, the evidence of a co-occurrence of deficits involving gesture execution and error recognition or awareness indicates that a specific system of action monitoring is involved in action. Although the results are only preliminary, since the potential experimental and clinical implications are significant, action recognition and error monitoring require in the future more in depth investigation.

## Conclusion

In this review, we have taken into account a good deal of recent evidence on the subject of the interaction between objects, body parts and the environment and have thus been able to use our findings to emphasize the important role that tools and objects play in the perception, understanding and production of actions. We suggest that the use of objects is the result of a multifaceted process where multiple components are involved. These include not only knowledge about an object and sensory-motor representations, but also spatial and body representations and executive functions. In addition, a specific system devoted to the monitoring of actions is probably necessary in order to check performance. Many questions remain unresolved, such as the role of the right hemisphere in apraxia and the importance of action monitoring system in awareness of errors. These issues need further in depth investigation in order to understand their potential impact in the definition of new models of motor controls and in the devising of new rehabilitative techniques.

## Author Contributions

LC and VM contributed to the concept of the article and prepared the first draft of the manuscript. All authors discussed, reviewed and contributed to the published version of the manuscript. MS contributed in preparing figures.

## Conflict of Interest Statement

The authors declare that the research was conducted in the absence of any commercial or financial relationships that could be construed as a potential conflict of interest.
